# Supplementary material for: A Novel Defined PANoptosis-Related miRNA Signature for Predicting the Prognosis and Immune Characteristics in Clear Cell Renal Cell Carcinoma: A miRNA Signature for the Prognosis of ccRCC
Source: Int J Mol Sci. 2023 May 28;24(11):9392. doi: 10.3390/ijms24119392 (PMC10253790; doi:10.3390/ijms24119392)
Supplement: Supplementary file 1 [file ijms-24-09392-s001.zip › Supplementary Figure.pdf]

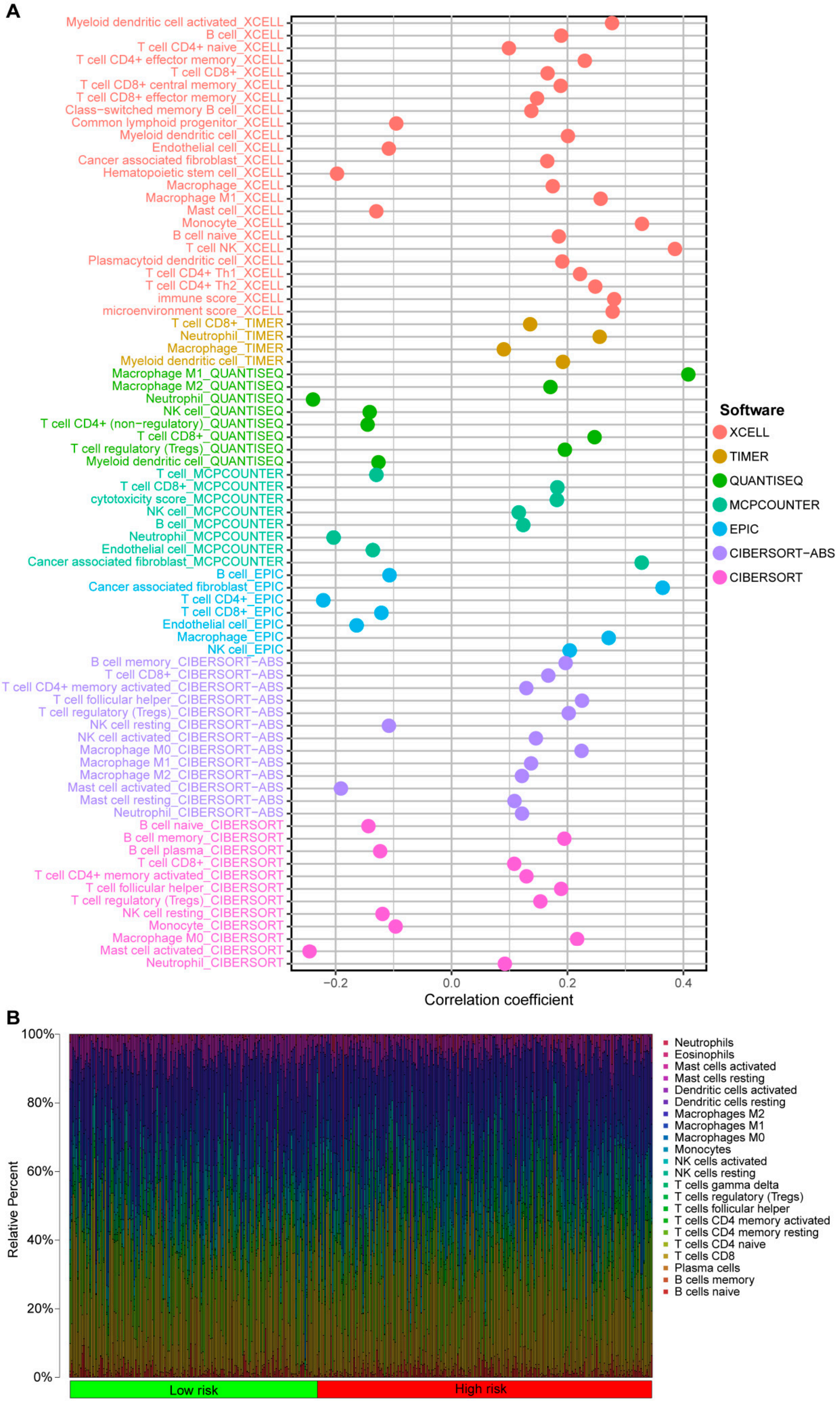
A
Myeloid dendritic cell activated_XCELL
B cell_XCELL
T cell CD4+ naive_XCELL
T cell CD4+ effector memory_XCELL
T cell CD8+_XCELL
T cell CD8+ central memory_XCELL
T cell CD8+ effector memory_XCELL
Class−switched memory B cell_XCELL
Common lymphoid progenitor_XCELL
Myeloid dendritic cell_XCELL
Endothelial cell_XCELL
Cancer associated fibroblast_XCELL
Hematopoietic stem cell_XCELL
Macrophage_XCELL
Macrophage M1_XCELL
Mast cell_XCELL
Monocyte_XCELL
B cell naive_XCELL
T cell NK_XCELL
Plasmacytoid dendritic cell_XCELL
T cell CD4+ Th1_XCELL
T cell CD4+ Th2_XCELL
immune score_XCELL
microenvironment score_XCELL
T cell CD8+_TIMER
Neutrophil_TIMER
Macrophage_TIMER
Myeloid dendritic cell_TIMER
Macrophage M1_QUANTISEQ
Macrophage M2_QUANTISEQ
Neutrophil_QUANTISEQ
NK cell_QUANTISEQ
T cell CD4+ (non−regulatory)_QUANTISEQ
T cell CD8+_QUANTISEQ
T cell regulatory (Tregs)_QUANTISEQ
Myeloid dendritic cell_QUANTISEQ
T cell_MCPCOUNTER
T cell CD8+_MCPCOUNTER
cytotoxicity score_MCPCOUNTER
NK cell_MCPCOUNTER
B cell_MCPCOUNTER
Neutrophil_MCPCOUNTER
Endothelial cell_MCPCOUNTER
Cancer associated fibroblast_MCPCOUNTER
B cell_EPIC
Cancer associated fibroblast_EPIC
T cell CD4+_EPIC
T cell CD8+_EPIC
Endothelial cell_EPIC
Macrophage_EPIC
NK cell_EPIC
B cell memory_CIBERSORT−ABS
T cell CD8+_CIBERSORT−ABS
T cell CD4+ memory activated_CIBERSORT−ABS
T cell follicular helper_CIBERSORT−ABS
T cell regulatory (Tregs)_CIBERSORT−ABS
NK cell resting_CIBERSORT−ABS
NK cell activated_CIBERSORT−ABS
Macrophage M0_CIBERSORT−ABS
Macrophage M1_CIBERSORT−ABS
Macrophage M2_CIBERSORT−ABS
Mast cell activated_CIBERSORT−ABS
Mast cell resting_CIBERSORT−ABS
Neutrophil_CIBERSORT−ABS
B cell naive_CIBERSORT
B cell memory_CIBERSORT
B cell plasma_CIBERSORT
T cell CD8+_CIBERSORT
T cell CD4+ memory activated_CIBERSORT
T cell follicular helper_CIBERSORT
T cell regulatory (Tregs)_CIBERSORT
NK cell resting_CIBERSORT
Monocyte_CIBERSORT
Macrophage M0_CIBERSORT
Mast cell activated_CIBERSORT
Neutrophil_CIBERSORT
−0.2
0.0
0.2
0.4
Correlation coefficient
Software
XCELL
TIMER
QUANTISEQ
MCPCOUNTER
EPIC
CIBERSORT−ABS
CIBERSORT
B
100%
80%
60%
40%
20%
0%
Relative Percent
Low risk
High risk
Neutrophils
Eosinophils
Mast cells activated
Mast cells resting
Dendritic cells activated
Dendritic cells resting
Macrophages M2
Macrophages M1
Macrophages M0
Monocytes
NK cells activated
NK cells resting
T cells gamma delta
T cells regulatory (Tregs)
T cells follicular helper
T cells CD4 memory activated
T cells CD4 memory resting
T cells CD4 naive
T cells CD8
Plasma cells
B cells memory
B cells naive

Supplementary Figure S1. The immune cell infiltration in TME.

(A) The correlation between risk score and immune cell infiltration in KIRC samples.

(B) The panorama showed the abundance of 22 types of immune cells used by the CIBERSORT analytical tool for different risk groups.

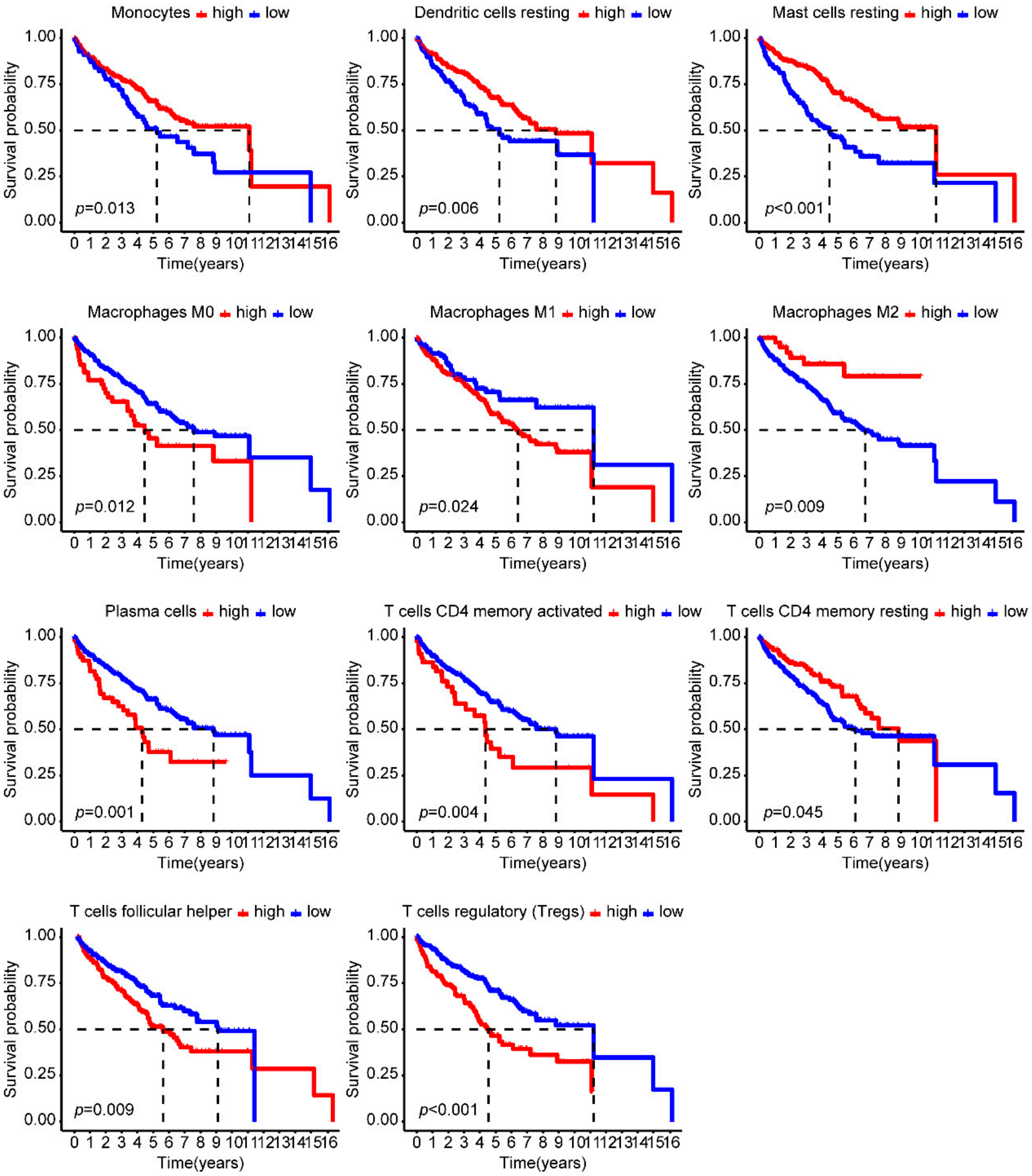

Supplementary Figure S2. The overall survival analysis for the immune cells from the CIBERSORT analysis.

The blue curve represents the low-infiltration group, and the red curve represents the high-infiltration group.

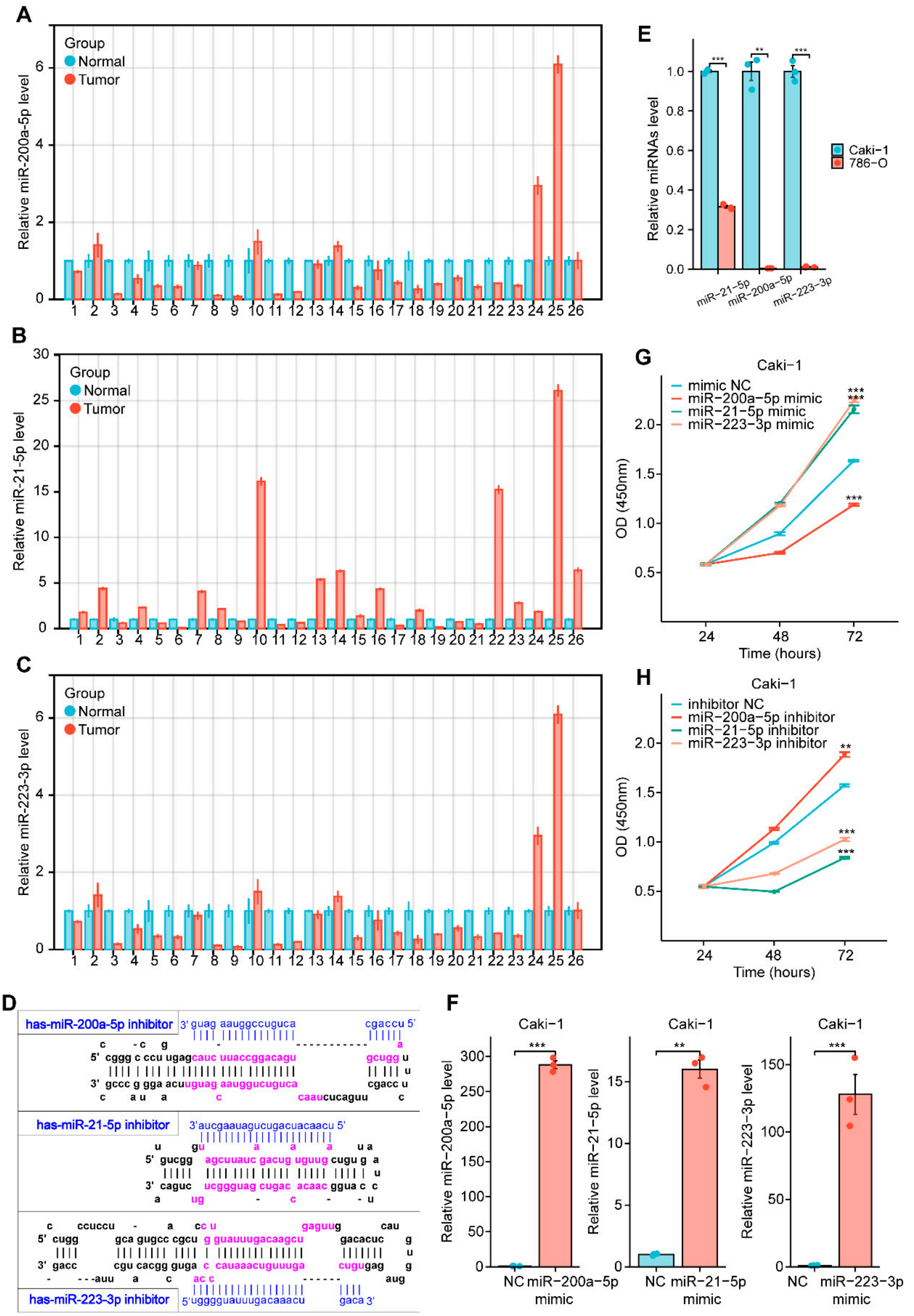

Supplementary Figure S3. Functional Enrichment Analysis of Distinctive miRNAs. (A-C) The relative expression ratio of miRNAs in 26 paired kidney tumor and adjacent normal tissue. (D) The design structure of miRNA inhibitors. (E) The relative expression level of miRNAs in Caki-1 and 786-O. (F) The transfection efficiency of

miRNAs mimics in Caki-1 cells. (G,H) CCK-8 assay of Caki-1 cell viability changes in 72 hours with miRNA mimics and inhibitors transfection. ** $p < 0.01$, *** $p < 0.001$.
