# Supplementary material for: A Novel Defined PANoptosis-Related miRNA Signature for Predicting the Prognosis and Immune Characteristics in Clear Cell Renal Cell Carcinoma: A miRNA Signature for the Prognosis of ccRCC
Source: Int J Mol Sci. 2023 May 28;24(11):9392. doi: 10.3390/ijms24119392 (PMC10253790; doi:10.3390/ijms24119392)
Supplement: Supplementary file 1 [file ijms-24-09392-s001.zip › Supplementary Table S1-literature reports.pdf]

Supplementary Table S1. Literature reports about PANoptosis-related miRNAs

miRNAs	Related functions reported in previous literature		
	**Pyroptosis**	**Apoptosis**	**Necroptosis**
miR-141-3p	MiR-141-3p repressed NLRP3-mediated pyroptosis by suppressing NLRP3, thus protecting calcium oxalate (CaOx) crystal-induced renal tubular epithelial cell (RTEC) injury [1].	The elevation of miR-141-3p resulted in the promotion of apoptosis in osteosarcoma cell lines [2] as well as in colon cancer cells [3]. However, miR-141-3p induces cell proliferation and apoptosis resistance via targeted binding with the 3'-UTR of the transcription factor kruppel-like factor-9 (KLF9) in prostate cancer cells [4].	The overexpression of miR-141-3p inhibited upregulation of necroptosis-related molecules and interaction of receptor interacting protein kinase 1 (RIPK1) and RIPK3 in LPS-treated Caco-2 cells for necrotizing enterocolitis [5].
miR-148a-3p	The silencing of LncRNA HOTTIP led to the inhibition of cell proliferation and NLRP1 inflammasome-mediated pyroptosis by negatively regulating miR-148a-3p targeting AKT2 and then increased ASK1/JNK signaling [6].	MiR-148a-3p was reported to promote cell apoptosis by targeting CDK6 in acute myeloid leukemia cells [7], ROCK-1 in bladder cancer [8], and SYNJ1 in the neuronal cells [9].	LncRNA-107053293 regulated necroptosis by acting as a competing endogenous RNA of miR-148a-3p in chicken trachea after NH3 inhalation [10].
miR-15a-5p	Inhibition of miR-15a increased inflammatory cytokines, activates caspase-1 inflammasome and increases Gasdermin D, an effector of pyroptosis in HUVECs by regulating SMPD1 [11].	MiR-15a-5p-targeting oncogene YAP1 inhibited cell viability and induced cell apoptosis in cervical cancer cells [12].	MiR-15a was up-regulated in tracheal tissue and LMH cells under H2S exposure, which triggered apoptosis and necroptosis [13], but LPS stimulation inhibited miR-15a, and increased the expression of JNK and necroptosis in chicken lungs [14].
miR-155-5p	Andrographolide (Andro) downregulated the expression of proinflammatory miR-155-5p, which then promoted the expression of Nrf2 to suppress pyroptosis in Mycobacterium tuberculosis (Mtb)-infected macrophages [15].	miR-155-5p promoted apoptosis through inhibition of autophagy in liver carcinoma cells [16] and inhibited gastric cancer-cell proliferation by targeting MAP3K10 [17].	MiR-155-5p was identified to suppress its target Ripk1 (receptor (TNFRSF)-interacting serine-threonine kinase 1) expression, which is directly involved in apoptosis/necroptosis in murine osteosarcoma models [18].
miR-16-5p	Puerarin alleviates oxidative stress and pyroptosis in high glucose-stimulated human retinal endothelial cells (HRECs) injury by regulating the miR-16-5p/CASP1 axis [19].	miR-16-5p induced apoptosis by regulating ANLN in breast cancer [20] and by targeting AKT3 in prostate cancer [21].	Selenium deficiency regulated the miR-16-5p-PI3K/AKT pathway and exacerbated LPS-induced necroptosis in chicken tracheal epithelial cells [22].
miR-181-5p	Chlorpyrifos could inhibit cell proliferation, and activate cell pyroptosis by elevating miR-181 through down-regulation of the SIRT1/PGC-1α/Nrf2 pathway in human neuroblastoma SH-SY5Y cells [23].	LncRNA SNHG5 may inhibit apoptosis of diffuse large B cell lymphoma cells via targeting miR-181-5p/XIAP [24].	Cadmium might act through miR-181-5p/TNF-α to induce necroptosis in swine lungs [25]. MiR-181-5p was also reported having a key role in necroptosis in carp lymphocytes exposed to Atrazine by downregulating the expression of HK and TNF-α [26].
miR-200a-5p	MiR-200a was degraded by IRE1α in diabetic nephropathy rats, and stimulated the TXINP/NLRP3 pathway-mediated pyroptosis and renal damage [27].	The overexpression of miR-200a facilitated apoptosis by targeting BRD4 in castration-resistant prostate cancer (CRPC) cells [28], promoted TRAIL-triggered apoptosis by inhibiting A20 in gastric cancer [29], elevated the apoptosis of Wilm's tumor cells by regulating CDC7 [30] and promoted apoptosis in non-small cell lung cancer cells [31].	The overexpression of miR-200a-5p sparked the receptor interacting serine/threonine kinase 3 (RIP3)-dependent necroptosis in vivo and in vitro via targeting RNF11 in heart disease [32].
miR-21-5p	MiR-21-5p overexpression upregulated pyroptosis-associated mRNAs and proteins by suppressing TGFBI in colorectal cancer [33] and via A20 in LPS-induced septic shock [34].	miR-21-5p inhibitor with Aurora kinase inhibitor reversine produced a synergistic effect to limit human breast cancer progression [35]. Otherwise, miR-21-5p down-regulation also inhibited cell proliferation and induced cell apoptosis in esophageal squamous cell carcinoma cells [36].	LncRNA SNHG1/miR-21-5p/TLR4 regulatory axis was identified in the progression in stomach adenocarcinoma [37].
miR-210-3p	Transfection of miR-210 mimics suppressed the effects of paeoniflorin on hypoxia-induced pyroptosis in astrocytes for hypoxia-induced brain injury in rats [38].	Ectopic expression of miR-210-3p reduced serum starvation-induced cell apoptosis in triple-negative breast cancer [39] and	Inhibition of Kras-derived exosomes downregulated immunosuppressive BACH2/GATA-3 expression via RIP-3/TNFa dependent necroptosis and miR-146/miR-210 modulation [41].

		suppressed apoptosis by targeting SIN3A of non-small cell lung cancer [40].	
miR-223-3p	MiR-223-3p inhibited pyroptosis in monosodium urate (MSU)-induced gout arthritis (GA) rats and fibroblast-like synoviocytes (FLSs) by targeting NLRP3 [42]. In addition, miR-223-3p in salivary exosomes might inhibit GSDMD-mediated pyroptosis by targeting NLRP3 in periodontitis [43]. Nevertheless, miR-233-3p was identified to promote pyroptosis of cardiomyocytes and release of inflammasome factors via downregulating the expression level of SPI1 [44].	MiR-223-3p served as an oncogenic role, which promotes cell growth and inhibits apoptosis through repression of FBXW7 in testicular germ cell tumors [45] and through targeting PRDM1 in colon cancer [46]. Besides, transfection with miR-223-3p inhibitor increased chemo-sensitivity to docetaxel and cell apoptosis rate in prostatic cancer [47].	MiR-223-3p was found to suppress RIP1/RIP3/MLKL necroptotic pathway by inhibiting NLRP3 in ischemic/reperfused hearts [48], by targeting the 3' un-translated region of RIPK3 in 3-MCPD-dipalmitate-induced acute kidney injury (AKI) [49], by negatively regulating RIP3 in spinal cord injury [50].
miR-29a-3p	miR-29a-3p was down-regulated in plasma of acute respiratory distress syndrome (ARDS) patients, and miR-29a-3p agomir injection reduced alveolar epithelial cell PANoptosis, ultimately improving lung injury in the acute lung injury (ALI) model mice [51].		

Reference

1. Gan, X. G.; Wang, Z. H.; Xu, H. T., Mechanism of miRNA-141-3p in Calcium Oxalate-Induced Renal Tubular Epithelial Cell Injury via NLRP3-Mediated Pyroptosis. *Kidney Blood Press Res* **2022,** *47* (5), 300-308.
2. Wang, N.; Li, P.; Liu, W.; Wang, N.; Lu, Z.; Feng, J.; Zeng, X.; Yang, J.; Wang, Y.; Zhao, W., miR-141-3p suppresses proliferation and promotes apoptosis by targeting GLI2 in osteosarcoma cells. *Oncol Rep* **2018,** *39* (2), 747-754.
3. Tong, S. J.; Zhang, X. Y.; Guo, H. F.; Yang, J.; Qi, Y. P.; Lu, S., Study on effects of miR-141-3p in proliferation, migration, invasion and apoptosis of colon cancer cells by inhibiting Bcl2. *Clin Transl Oncol* **2021,** *23* (12), 2526-2535.
4. Li, J. Z.; Li, J.; Wang, H. Q.; Li, X.; Wen, B.; Wang, Y. J., MiR-141-3p promotes prostate cancer cell proliferation through inhibiting kruppel-like factor-9 expression. *Biochemical and biophysical research communications* **2017,** *482* (4), 1381-1386.
5. Li, X.; Wang, Y.; Wang, Y.; He, X., MiR-141-3p ameliorates RIPK1-mediated necroptosis of intestinal epithelial cells in necrotizing enterocolitis. *Aging (Albany NY)* **2020,** *12* (18), 18073-18083.
6. Tan, C.; Liu, W.; Zheng, Z. H.; Wan, X. G., LncRNA HOTTIP inhibits cell pyroptosis by targeting miR-148a-3p/AKT2 axis in ovarian cancer. *Cell Biol Int* **2021,** *45* (7), 1487-1497.
7. Zhou, H.; Jia, X.; Yang, F.; Shi, P., miR-148a-3p suppresses the progression of acute myeloid leukemia via targeting cyclin-dependent kinase 6 (CDK6). *Bioengineered* **2021,** *12* (1), 4508-4519.
8. Xu, C.; Zhou, G.; Sun, Z.; Zhang, Z.; Zhao, H.; Jiang, X., miR-148a-3p inhibits the proliferation and migration of bladder cancer via regulating the expression of ROCK-1. *PeerJ* **2022,** *10*, e12724.
9. Xu, J.; Sun, M.; Li, X.; Huang, L.; Gao, Z.; Gao, J.; Xie, A., MicroRNA expression profiling after recurrent febrile seizures in rat and emerging role of miR-148a-3p/SYNJ1 axis. *Sci Rep* **2021,** *11* (1), 1262.
10. Wang, W.; Shi, Q.; Wang, S.; Zhang, H.; Xu, S., Ammonia regulates chicken tracheal cell necroptosis via the LncRNA-107053293/MiR-148a-3p/FAF1 axis. *J Hazard Mater* **2020,** *386*, 121626.
11. Rana, S.; Espinosa-Diez, C.; Ruhl, R.; Chatterjee, N.; Hudson, C.; Fraile-Bethencourt, E.; Agarwal, A.; Khou, S.; Thomas, C. R., Jr.; Anand, S., Differential regulation of microRNA-15a by radiation affects angiogenesis and tumor growth via modulation of acid sphingomyelinase. *Sci Rep* **2020,** *10* (1), 5581.

12. Chen, X.; Cao, R.; Liu, H.; Zhang, T.; Yuan, X.; Xu, S., MicroRNA15a5ptargeting oncogene YAP1 inhibits cell viability and induces cell apoptosis in cervical cancer cells. *Int J Mol Med* **2020,** *46* (4), 1301-1310.
13. Li, X.; Chen, M.; Shi, Q.; Zhang, H.; Xu, S., Hydrogen sulfide exposure induces apoptosis and necroptosis through lncRNA3037/miR-15a/BCL2-A20 signaling in broiler trachea. *Sci Total Environ* **2020,** *699*, 134296.
14. Wang, B.; Cui, Y.; Zhang, Q.; Wang, S.; Xu, S., Selenomethionine alleviates LPS-induced JNK/NLRP3 inflammasome-dependent necroptosis by modulating miR-15a and oxidative stress in chicken lungs. *Metallomics* **2021,** *13* (8).
15. Fu, Y.; Shen, J.; Liu, F.; Zhang, H.; Zheng, Y.; Jiang, X., Andrographolide Suppresses Pyroptosis in Mycobacterium tuberculosis-Infected Macrophages via the microRNA-155/Nrf2 Axis. *Oxid Med Cell Longev* **2022,** *2022*, 1885066.
16. Yu, Q.; Xu, X. P.; Yin, X. M.; Peng, X. Q., miR-155-5p increases the sensitivity of liver cancer cells to adriamycin by regulating ATG5-mediated autophagy. *Neoplasma* **2021,** *68* (1), 87-95.
17. Li, S.; Zhang, T.; Zhou, X.; Du, Z.; Chen, F.; Luo, J.; Liu, Q., The tumor suppressor role of miR-155-5p in gastric cancer. *Oncol Lett* **2018,** *16* (2), 2709-2714.
18. Bhattacharya, S.; Chalk, A. M.; Ng, A. J.; Martin, T. J.; Zannettino, A. C.; Purton, L. E.; Lu, J.; Baker, E. K.; Walkley, C. R., Increased miR-155-5p and reduced miR-148a-3p contribute to the suppression of osteosarcoma cell death. *Oncogene* **2016,** *35* (40), 5282-5294.
19. Zhang, J.; Chen, Y.; Gao, W., Puerarin protects against human retinal endothelial cells injury induced by high glucose via regulating miR-16-5p/CASP1 axis. *Gen Physiol Biophys* **2021,** *40* (3), 235-243.
20. Wang, Z.; Hu, S.; Li, X.; Liu, Z.; Han, D.; Wang, Y.; Wei, L.; Zhang, G.; Wang, X., MiR-16-5p suppresses breast cancer proliferation by targeting ANLN. *BMC Cancer* **2021,** *21* (1), 1188.
21. Wang, F.; Wang, W.; Lu, L.; Xie, Y.; Yan, J.; Chen, Y.; Di, C.; Gan, L.; Si, J.; Zhang, H.; Mao, A., MicroRNA165p regulates cell survival, cell cycle and apoptosis by targeting AKT3 in prostate cancer cells. *Oncol Rep* **2020,** *44* (3), 1282-1292.
22. Wang, L.; Shi, X.; Zheng, S.; Xu, S., Selenium deficiency exacerbates LPS-induced necroptosis by regulating miR-16-5p targeting PI3K in chicken tracheal tissue. *Metallomics* **2020,** *12* (4), 562-571.
23. Zhao, M. W.; Yang, P.; Zhao, L. L., Chlorpyrifos activates cell pyroptosis and increases susceptibility on oxidative stress-induced toxicity by miR-181/SIRT1/PGC-1alpha/Nrf2 signaling pathway in human neuroblastoma SH-SY5Y cells: Implication for association between chlorpyrifos and Parkinson's disease. *Environ Toxicol* **2019,** *34* (6), 699-707.
24. Xing, X.; Xu, T.; Liu, B.; Guo, Q., LncRNA SNHG5 can Regulate the Proliferation and Migration of Diffuse Large B Cell Lymphoma Progression via Targeting miR-181-5p/XIAP. *Journal of Cancer* **2022,** *13* (3), 784-792.
25. Zhang, W.; Sun, X.; Shi, X.; Qi, X.; Shang, S.; Lin, H., Subacute Cadmium Exposure Induces Necroptosis in Swine Lung via Influencing Th1/Th2 Balance. *Biol Trace Elem Res* **2022**.
26. Cui, Y.; Yin, K.; Gong, Y.; Qu, Y.; Liu, H.; Lin, H., Atrazine induces necroptosis by miR-181-5p targeting inflammation and glycometabolism in carp lymphocytes. *Fish Shellfish Immunol* **2019,** *94*, 730-738.
27. Ke, R.; Wang, Y.; Hong, S.; Xiao, L., Endoplasmic reticulum stress related factor IRE1α regulates TXNIP/NLRP3-mediated pyroptosis in diabetic nephropathy. *Experimental cell research* **2020,** *396* (2), 112293.

28. Guan, H.; You, Z.; Wang, C.; Fang, F.; Peng, R.; Mao, L.; Xu, B.; Chen, M., MicroRNA-200a suppresses prostate cancer progression through BRD4/AR signaling pathway. *Cancer medicine* **2019,** *8* (4), 1474-1485.
29. Guo, T.; Zhang, Y.; Qu, X.; Che, X.; Li, C.; Fan, Y.; Wan, X.; Ma, R.; Hou, K.; Zhou, H.; He, X.; Hu, X.; Liu, Y.; Xu, L., miR-200a enhances TRAIL-induced apoptosis in gastric cancer cells by targeting A20. *Cell Biol Int* **2018,** *42* (5), 506-514.
30. Liang, X. L.; Wang, Y. L.; Wang, P. R., MiR-200a with CDC7 as a direct target declines cell viability and promotes cell apoptosis in Wilm's tumor via Wnt/β-catenin signaling pathway. *Molecular and cellular biochemistry* **2021,** *476* (6), 2409-2420.
31. Huang, Y.; Bao, T.; Li, Z.; Ji, G.; Zhang, L., Function of miR-200a in proliferation and apoptosis of non-small cell lung cancer cells. *Oncol Lett* **2020,** *20* (2), 1256-1262.
32. Yang, T.; Cao, C.; Yang, J.; Liu, T.; Lei, X. G.; Zhang, Z.; Xu, S., miR-200a-5p regulates myocardial necroptosis induced by Se deficiency via targeting RNF11. *Redox Biol* **2018,** *15*, 159-169.
33. Jiang, R.; Chen, X.; Ge, S.; Wang, Q.; Liu, Y.; Chen, H.; Xu, J.; Wu, J., MiR-21-5p Induces Pyroptosis in Colorectal Cancer via TGFBI. *Front Oncol* **2020,** *10*, 610545.
34. Xue, Z.; Xi, Q.; Liu, H.; Guo, X.; Zhang, J.; Zhang, Z.; Li, Y.; Yang, G.; Zhou, D.; Yang, H.; Zhang, L.; Zhang, Q.; Gu, C.; Yang, J.; Da, Y.; Yao, Z.; Duo, S.; Zhang, R., miR-21 promotes NLRP3 inflammasome activation to mediate pyroptosis and endotoxic shock. *Cell Death Dis* **2019,** *10* (6), 461.
35. Zhang, Y.; Wang, Y.; Xue, J.; Liang, W.; Zhang, Z.; Yang, X.; Qiao, Z.; Jiang, Y.; Wang, J.; Cao, X.; Chen, P., Co-treatment with miR-21-5p inhibitor and Aurora kinase inhibitor reversine suppresses breast cancer progression by targeting sprouty RTK signaling antagonist 2. *Bioengineered* **2022,** *13* (1), 455-468.
36. Li, X.; Chen, D.; Li, M.; Gao, X.; Shi, G.; Zhao, H., The CADM2/Akt pathway is involved in the inhibitory effect of miR-21-5p downregulation on proliferation and apoptosis in esophageal squamous cell carcinoma cells. *Chemico-biological interactions* **2018,** *288*, 76-82.
37. Wang, N.; Liu, D., Identification and Validation a Necroptosis-related Prognostic Signature and Associated Regulatory Axis in Stomach Adenocarcinoma. *OncoTargets and therapy* **2021,** *14*, 5373-5383.
38. Jiang, Z.; Chen, J.; Chen, J.; Lei, Z.; Chen, H.; Wu, J.; Bai, X.; Wanyan, P.; Yu, Q., Anti-inflammatory effects of paeoniflorin caused by regulation of the hif1a/miR-210/caspase1/GSDMD signaling pathway in astrocytes: a novel strategy for hypoxia-induced brain injury in rats. *Immunopharmacol Immunotoxicol* **2021,** *43* (4), 410-418.
39. Du, Y.; Wei, N.; Ma, R.; Jiang, S.; Song, D., A miR-210-3p regulon that controls the Warburg effect by modulating HIF-1α and p53 activity in triple-negative breast cancer. *Cell Death Dis* **2020,** *11* (9), 731.
40. Ren, J.; Li, X.; Dong, H.; Suo, L.; Zhang, J.; Zhang, L.; Zhang, J., miR-210-3p regulates the proliferation and apoptosis of non-small cell lung cancer cells by targeting SIN3A. *Exp Ther Med* **2019,** *18* (4), 2565-2573.
41. Petanidis, S.; Domvri, K.; Porpodis, K.; Anestakis, D.; Freitag, L.; Hohenforst-Schmidt, W.; Tsavlis, D.; Zarogoulidis, K., Inhibition of kras-derived exosomes downregulates immunosuppressive BACH2/GATA-3 expression via RIP-3 dependent necroptosis and miR-146/miR-210 modulation. *Biomed Pharmacother* **2020,** *122*, 109461.

42. Tian, J.; Zhou, D.; Xiang, L.; Liu, X.; Zhang, H.; Wang, B.; Xie, B., MiR-223-3p inhibits inflammation and pyroptosis in monosodium urate-induced rats and fibroblast-like synoviocytes by targeting NLRP3. *Clin Exp Immunol* **2021,** *204* (3), 396-410.
43. Xia, Y.; Zhou, K.; Sun, M.; Shu, R.; Qian, J.; Xie, Y., The miR-223-3p Regulates Pyroptosis Through NLRP3-Caspase 1-GSDMD Signal Axis in Periodontitis. *Inflammation* **2021,** *44* (6), 2531-2542.
44. Zhao, S.; Tan, Y.; Qin, J.; Xu, H.; Liu, L.; Wan, H.; Zhang, C.; Fan, W.; Qu, S., MicroRNA-223-3p promotes pyroptosis of cardiomyocyte and release of inflammasome factors via downregulating the expression level of SPI1 (PU.1). *Toxicology* **2022,** *476*, 153252.
45. Richie, J. P., Re: miR-223-3p Regulates Cell Growth and Apoptosis via FBXW7 Suggesting an Oncogenic Role in Human Testicular Germ Cell Tumors. *J Urol* **2018,** *199* (5), 1114.
46. Chai, B.; Guo, Y.; Cui, X.; Liu, J.; Suo, Y.; Dou, Z.; Li, N., MiR-223-3p promotes the proliferation, invasion and migration of colon cancer cells by negative regulating PRDM1. *Am J Transl Res* **2019,** *11* (7), 4516-4523.
47. Feng, Q.; He, P.; Wang, Y., MicroRNA-223-3p regulates cell chemo-sensitivity by targeting FOXO3 in prostatic cancer. *Gene* **2018,** *658*, 152-158.
48. Qin, D.; Wang, X.; Li, Y.; Yang, L.; Wang, R.; Peng, J.; Essandoh, K.; Mu, X.; Peng, T.; Han, Q.; Yu, K. J.; Fan, G. C., MicroRNA-223-5p and -3p Cooperatively Suppress Necroptosis in Ischemic/Reperfused Hearts. *J Biol Chem* **2016,** *291* (38), 20247-59.
49. Huang, G.; Xue, J.; Sun, X.; Wang, J.; Yu, L. L., Necroptosis in 3-chloro-1, 2-propanediol (3-MCPD)-dipalmitate-induced acute kidney injury in vivo and its repression by miR-223-3p. *Toxicology* **2018,** *406-407*, 33-43.
50. Wang, Y.; Jiao, J.; Ren, P.; Wu, M., Upregulation of miRNA-223-3p ameliorates RIP3-mediated necroptosis and inflammatory responses via targeting RIP3 after spinal cord injury. *J Cell Biochem* **2019**.
51. Cui, Y.; Wang, X.; Lin, F.; Li, W.; Zhao, Y.; Zhu, F.; Yang, H.; Rao, M.; Li, Y.; Liang, H.; Dai, M.; Liu, B.; Chen, L.; Han, D.; Lu, R.; Peng, W.; Zhang, Y.; Song, C.; Luo, Y.; Pan, P., MiR-29a-3p Improves Acute Lung Injury by Reducing Alveolar Epithelial Cell PANoptosis. *Aging Dis* **2022,** *13* (3), 899-909.
